# Cost analysis favours SPECT over PET and CTA for evaluation of coronary artery disease: the SPARC study

**DOI:** 10.1007/s12471-014-0558-4

**Published:** 2014-04-23

**Authors:** E. E. van der Wall

**Affiliations:** Interuniversity Cardiology Institute of the Netherlands (ICIN)-Netherlands Heart Institute, Catherijnesingel 52, P.O. Box 19258, 3501 DG Utrecht, the Netherlands

There are many ways to perform noninvasive testing in patients with coronary artery disease (CAD), including myocardial perfusion imaging with single-photon emission computed tomography (SPECT), positron-emission tomography (PET), and coronary computed tomography angiography (CTA). Results from series of patients evaluated with single modalities have already been published by many researchers [1–3], but there are few head-to-head comparisons of the outcomes of alternative test strategies [4, 5]. Consequently, the comparative effectiveness of different noninvasive cardiac testing strategies has been difficult to assess.

The recently published SPARC registry (Study of Myocardial Perfusion and Coronary Anatomy Imaging Roles in Coronary Artery Disease) in JACC by the group of DiCarli et al. (Stanford, CA, USA) was a multicentre study designed to collect standardised clinical data on patients undergoing CTA, PET, or SPECT [6]. The primary purpose of the SPARC study was to evaluate the economic outcomes of using CTA, PET, or SPECT to evaluate patients with suspected CAD. The authors used an observational registry of 1703 patients: 590 patients underwent CTA, 548 patients had PET and 565 patients SPECT for diagnosis of suspected CAD. Patients were followed for 2 years; documented resource use, medical costs for CAD, and clinical outcomes were assessed.

The main findings were that costs were significantly lower for SPECT compared with CTA or PET in the evaluation of suspected CAD. SPECT was economically attractive compared with PET, whereas CTA was associated with higher costs and no significant difference in mortality compared with SPECT. More specifically, the authors observed that patients who underwent coronary CTA and PET experienced higher rates of subsequent invasive coronary arteriography (16 % and 15 %, respectively) as compared with patients who underwent SPECT (7 %). The mean total radiation was significantly higher for CTA (15.1 mSV) compared with SPECT (11.7 mSV), both for the initial tests (13 mSV vs. 11 mSV) and for follow-up tests and procedures (2.1 mSV vs. 0.7 mSV). Exposure from follow-up tests and procedures was higher after PET (2.0 mSV) than after SPECT (0.6 mSV).

The findings in favour of SPECT are in line with a decision model by Garber and Solomon [7], who compared exercise treadmill testing, stress echocardiography, planar thallium imaging, SPECT, and PET imaging with a strategy of immediate invasive angiography. They found SPECT to be much more cost-effective than PET for noninvasive diagnosis, and SPECT to be a better option than immediate invasive coronary angiography. In terms of outcomes, Shaw et al. [1] found that patients who underwent initial SPECT with selective cardiac catheterisation had lower costs than patients who underwent routine coronary angiography (END study). In the EMPIRE study by Underwood et al. [2] it was demonstrated that strategies using myocardial perfusion imaging were cheaper and equally effective when compared with strategies that did not use myocardial perfusion imaging, both for the cost of diagnosis and for overall 2-year management costs; the 2-year patient outcome was the same. Sharples et al. [8] randomised 898 patients to SPECT, stress echocardiography, magnetic resonance imaging (MRI), or direct invasive coronary angiography, and found SPECT to be as useful as immediate invasive angiography with similar costs. A limitation of the SPARC study is the fact that MRI was not included in the comparative strategies. In addition, as the study started in 2006, in recent years many technological revolutions have taken place for all imaging modalities indicating that studies such as SPARC should be repeated and extended every 5–10 years [9].

Analysis of the current SPARC registry at least suggests that SPECT is the noninvasive imaging procedure of choice to evaluate patients with suspected CAD in terms of subsequent medical costs. SPECT offered the lowest costs over 2 years of follow-up among patients with suspected CAD. The significantly higher costs among patients undergoing CTA or PET were primarily due to higher rates of subsequent invasive cardiac procedures, because there was little difference in initial costs of testing.

To conclude, the use of SPECT myocardial perfusion imaging was associated with lower costs over 2 years of follow-up than the use of CTA or PET, primarily because of fewer subsequent invasive procedures. Consequently, SPECT imaging seems to be the preferred imaging strategy in patients with suspected CAD. The value of SPECT imaging has been underscored by several recent reports [10–13]. However, larger and more definitive studies, such as the ongoing PROMISE (Prospective Multicenter Imaging Study for Evaluation of Chest Pain) trial (NCT01174550), are needed to assess the value and cost effectiveness of the various noninvasive imaging strategies.
